# Idiopathic Recurrent Polyneuritis Cranialis: A Rare Entity

**DOI:** 10.7759/cureus.4488

**Published:** 2019-04-17

**Authors:** Alcy R Torres, Carla Salvador, Mauricio Mora, Sharam Mirchandani, Wilson Chavez

**Affiliations:** 1 Pediatrics, Boston University School of Medicine, Boston, USA; 2 Pediatrics, Boston Universtiy School of Medicine, Boston, USA; 3 Radiology, Boston Medical Center, Boston, USA

**Keywords:** cranial nerve palsy, recurrent, ophthalmoplegia, bell’s palsy, polyneuritis

## Abstract

Recurrent polyneuritis cranialis is a rare disorder that can affect multiple cranial nerves. We describe a young man who presented with recurrent cranial nerve (CN) palsies. His first episode at 17 years of age involved the right VI cranial nerve, the second episode at age 21 involved the left V and VII cranial nerves while the last episode six months later affected the left IV cranial nerve. Based on the clinical findings and laboratory test results to exclude other possibilities, a diagnosis of idiopathic recurrent polyneuritis cranialis was made. This is a very rare disorder in childhood and adolescence. This is the youngest patient ever reported with recurrent polyneuritis cranialis of unknown etiology.

## Introduction

Polyneuritis cranialis (PNC) is a rare disorder affecting multiple cranial nerves [1]. Usually, it presents with a gradual and slowly progressive course [2]. The nerves usually affected in polyneuritis cranialis are the IV, V, VI, and VII nerves [1,3,4]. It is often attributed to prior viral infection, immune-mediated or idiopathic mechanism of injury [3]. It has been associated with Guillain-Barré syndrome, but this is an extremely rare presentation without any peripheral nerve involvement or ataxia [1,2,4]. The incidence of PNC remains unknown, and the rareness of the disease has made it more difficult to characterize it [1]. Its etiology has been attributed to many categories such as inflammatory, infectious, autoimmune, toxins, medications, vitamin deficiency, as well as idiopathic. Several conditions that have been attributed to inflammatory causes, including Guillain-Barré syndrome (GBS), a heterogeneous syndrome with several variant forms in both children and adults [4,5]; infections like Lyme disease, Chagas disease, diphtheria, varicella zoster virus (VZV), and botulism [4,5]; autoimmune disorders like myasthenia gravis [1], eosinophilic granulomatosis with polyangiitis [2,6], inflammatory bowel disease, sarcoidosis, and systemic lupus erythematosus [4,7]; Tolosa-Hunt syndrome [4,8]; toxins like arsenic, lead, organophosphorus esters; medications such as antibiotics (sulfonamide, chloramphenicol, metronidazole, and isoniazid) [9]; chemotherapeutic agents like vincristine, cytarabine [10]; vitamin deficiencies (commonly vitamin B6 or B12) [11]; and ophthalmoplegic migraine have been previously associated with polyneuritis cranialis [12].

It is thought that the pathological mechanism is related to microinfarction of cranial nerves due to occlusion of the vasa nervorum [3]. The syndrome usually, but not always, consists of a long prodromal headache and facial pain of subacute onset preceding the abrupt onset of multiple cranial nerve palsies [13]. An IRB approval was waived because of the nature of this publication.

## Case presentation

First event

A 17-year-old male presented for evaluation with an episode of horizontal diplopia to his doctor in his home country. He was "unable to move his right eye outwards." He did not complain of ear pain, headaches, nausea, neck pain, or vomiting. His symptoms resolved spontaneously in two to four weeks. Despite a thorough workup, no etiology was found and a diagnosis of right cranial nerve (CN) VI nerve palsy was made.

Second event

At the age of 21, the same patient presented with a left facial droop, decreased sensation on the left side of the face, drooling while drinking liquids, and a diagnosis of left V and VII cranial nerve palsy was made. At the time he complained of nasal congestion, coughing, and fatigue. He had no diaphoresis, fever, anorexia, headaches, nausea, abdominal pain, bowel pattern changes, chest pain, chills, arthralgia, joint swelling, myalgia, neck pain, rashes, sore throat, swollen glands, urinary symptoms, vertigo, visual changes, or vomiting.

On examination, his vital signs were normal. On general exam, he appeared well-developed, well-nourished and in no apparent distress. Neurological examination showed normal mental status, no meningeal signs, and no focalities except for facial asymmetry, and weakness involving the eyebrow, upper eyelid as well as decreased sensation across all three branches of the trigeminal nerve (Table 1).

**Table 1:** Test Results in the Second episode CBC: Complete Blood Count; ESR: Erythrocyte Sedimentation Rate; CRP: C-Reactive Protein; TSH: Thyroid-Stimulating Hormone; T4: Thyroxine; ANA: Antinuclear Antibody

Test Performed	Result
Lyme (B.burgdorferi) IgG, IgM EIA reflex wb	Negative
Comprehensive Respiratory Panel, PCR	Negative (Not detected)
CBC and differential, ESR, CRP	Normal
Basic Metabolic Panel	Normal
TSH, T4	Normal
ANA screening	Negative

He was diagnosed with Bell’s palsy and treated with prednisone for five days with complete resolution of symptoms.

Third event

He returned to the emergency room (ER) six months later with a sudden episode of double vision. He had some upper respiratory symptoms four days prior to the onset and was treated with amoxicillin-clavulanic acid. He had no associated pain, history of recent head or neck trauma, numbness or tingling of the face, facial asymmetry, or trouble swallowing or with speech. He denied visual aura, decreased vision, or other visual complaints. There was no diurnal fluctuation. The general exam was normal. His speech was fluent without any paraphasic errors and his comprehension was intact. The cranial nerves were intact except for left IV cranial nerve palsy.

Given his recent history of sinusitis, cavernous sinus thrombosis was considered but magnetic resonance imaging/ magnetic resonance venography (MRI/MRV) did not show evidence of thrombosis, demyelinating condition, tumor or bleeding. It only showed a Rathke cyst in the pituitary gland that was not compressive of any anatomic areas and was thought to be incidental. He was diagnosed with left IV cranial nerve palsy.

Past medical history revealed hypertension treated with enalapril with good control. He had traveled to Colorado, USA twice in the last two years to visit his relatives. There were no risk factors for HIV.

Extensive workup was done, including tests for Lyme (B.burgdorferi) IgG, IgM by Elisa and Western blot, comprehensive respiratory panel, complete blood count (CBC) and differential, erythrocyte sedimentation rate (ESR), C-reactive protein (CRP), basic metabolic panel, thyroid-stimulating hormone (TSH), T4, antinuclear antibody (ANA) screen, AChR Ab, angiotensin-converting enzyme (ACE), muscle-specific kinase (MuSK), hepatitis C virus (HCV), HIV, varicella zoster virus (VZV) antibody IgM and IgG, interferon-gamma release assays (IGRAs), chest X-Ray (CXR), head MRI and cerebrospinal fluid exam (CSF) (Tables 2-4).

**Table 2:** Test Results at the Third episode CBC: Complete Blood Count; ESR: Erythrocyte Sedimentation Rate; CRP: C-Reactive Protein; TSH: Thyroid-Stimulating Hormone; T4: Thyroxine; ANA: Antinuclear Antibody; CXR: Chest X-Ray; HIV: Human Immunodeficiency Virus

Test Performed	Result
CBC and differential, ESR, CRP	Within normal limits
Basic Metabolic Panel	Within normal limits
TSH, T4	Within normal limits
ANA screen	Negative
Acetylcholine receptor blocking antibody	Negative
Acetylcholine receptor modulating antibody	Negative
Acetylcholine receptor, binding	Negative
Angiotensin-converting enzyme	Negative
Quantiferon(r)-TB gold (client incubated)	Positive
CXR	Negative
HIV Ag/Ab combined assay	Negative
Varicella-zoster IgG	3.97 (ISR > or = 1.10 Positive)
Varicella-zoster virus antibody (IgM)	2.18 (ISR > or = 1.10 Positive)
Epstein Barr Virus (EBV) Antibody Panel	Negative
Trypanosoma cruzi antibody, total	Negative
Ehrlichia chaffeensis antibodies (IgG, IgM)	Negative
Rickettsia (rmsf) Abs (IgG,IgM) w/reflex to titers	Negative

**Table 3:** Cerebrospinal Fluid Exam: Gram Stain and PCR at the Third episode CSF: Cerebrospinal Fluid; RBC: Red Blood Cell; PCR: Polymerase Chain Reaction

Test Performed	Result
CSF Culture With Stat Gram Stain	6 RBCs, 1 nucleated cell, protein 27, glucose 58.
West Nile Virus IgG CSF	POSITIVE
West Nile Virus IgM CSF	NEGATIVE

**Table 4:** Meningitis/Encephalitis Panel, CSF, PCR at the Third episode CSF: Cerebrospinal Fluid; RBC: Red Blood Cell; PCR: Polymerase Chain Reaction

Test Performed	Result
Escherichia coli K1	NOT DETECTED
Haemophilus influenzae	NOT DETECTED
Listeria monocytogenes	NOT DETECTED
Neisseria meningitides (encapsulated)	NOT DETECTED
Streptococcus agalactiae	NOT DETECTED
Streptococcus pneumoniae	NOT DETECTED
Cytomegalovirus	NOT DETECTED
Enterovirus	NOT DETECTED
Herpes simplex virus 1	NOT DETECTED
Herpes simplex virus 2	NOT DETECTED
Human herpesvirus 6	NOT DETECTED
Human parechovirus	NOT DETECTED
Varicella-zoster virus	NOT DETECTED
Cryptococcus neoformans/gattii	NOT DETECTED

Varicella-zoster IgG and IgM antibodies were positive twice, with IgG antibody level being 3.97 and IgM 2.18 (ISR > or = 0.9 Positive). The antibody levels were followed and a gradual decrease in IgM levels over the next five months was noticed (2.18, 2.06, 1.38, 1.22). This time he did not receive any treatment; however, his symptoms improved spontaneously. Furthermore, varicella zoster virus (VZV) PCR was negative in the CSF.

## Discussion

In 1970, Steele and Vasuvat published an article claiming to have seen 14 adults with recurrent multiple cranial nerve palsies; however, out of the 14 patients, 12 were non-recurrent and none presented in the same pattern or the sequence of our patient. There were descriptions of only four cases in the report with details. The rest of the patients presented with different pathologies that ultimately led to secondary cranial neuropathies [1]. One out of two patients who had a relapse was initially diagnosed with optic neuritis.

Yagnik and Dhaduk reported polyneuritis cranialis as a complication of Lyme’s disease [14] and it has also been quoted as a manifestation subtype of GBS [15,16], but it was non-recurrent in both cases.

The preservation of tendon reflexes in idiopathic cranial neuropathy is the major clinical feature to distinguish PNC from GBS [2,5,15]. Our patient did not have any abnormality in his deep tendon reflex in upper and lower limbs.

Myasthenia gravis (MG) must be considered as well when a patient presents with sudden symptoms of diplopia, hence a measurement of anti-acetylcholine receptor antibody (blocking and binding) was performed and an extensive workup for myasthenia was negative; however MG is characterized by a steady progression to complete ophthalmoplegia or by relapses and remissions, and typically affects both eyes (ptosis) rather than one [17]. In our patient, only the left IV cranial nerve was involved.

Botulism was also considered in the differential diagnoses; however, affected patients subsequently developed generalized weakness [2]. Tolosa-Hunt syndrome is caused by an idiopathic granulomatous inflammation of the cavernous sinus and shares many features with PNC, like cranial nerve palsies, III, IV and first division of V [2,4,8]. It was considered because of the ophthalmoplegia [2,4,8], but because he had no pain and his MRI was negative, this syndrome was ruled out. Sarcoidosis which is a chronic systemic inflammatory disorder that is characterized by the formation of noncaseating granuloma may present with cranial nerve VI and VII palsies commonly [4]. In our patient a negative Angiotensin-converting enzyme (ACE) test, a normal chest X-ray and non-clinical findings suggestive of this disease allowed us to rule it out.

Typically, polyneuritis cranialis is preceded by a long prodromal headache and/or facial pain of subacute onset. Lee suggested that a headache should be considered as an additional cardinal feature of PNC along with multiple cranial neuropathies [1]. Ophthalmoplegic migraine was considered due to the fact that cranial nerves V and VII are usually affected in ophthalmoplegic migraine and a transitory ocular motor palsy that sometimes lasts as long as four weeks have been also reported [2], even though a headache was not the first symptom and was not prominent in any of the episodes.

Dosunmu indicated that most isolated fourth nerve palsies are congenital, even those presenting in adulthood [4,18,19]. The most common causes of acquired lesions are trauma and microvascular disease [4,18,19].

Polyneuritis cranialis has been described in conjunction with various conditions (Table 5).

**Table 5:** Differential Diagnosis ANA: Antinuclear Antibody; SLE: Systemic Lupus Erythematosus; ACE: Angiotensin-converting Enzyme; HIV: Human Immunodeficiency Virus; TB: Tuberculosis; IGRA: Interferon-Gamma Release Assay

Differential diagnosis	Other supporting findings	Diagnostic tests
Guillain-Barre syndrome	Ascending muscle paralysis	CSF-cytoalbuminologic dissociation
Myasthenia gravis	Muscle weakness, fatigue	Anti-Acetylcholine receptor antibody
Lyme disease	Fever, rash	Anti-B.burgdorferi IgM and IgG antibodies
Autoimmune conditions	Fever, joint pain, rash, etc.	ANA profile (SLE), ACE level (sarcoidosis)
Vitamin deficiency	Paresthesias, fatigue	Serum levels
Toxins	History of chronic medication consumption/chemotherapy	Liver and renal function tests
Other infections (HIV, TB)	Risk factors, weight loss, fever, etc, hemoptysis	HIV antigen/antibody assay, IGRAs

A preliminary diagnosis of varicella-zoster virus (VZV) was made based on the clinical presentation and positive IgM and IgG antibody levels in two separate occasions. A study by Yeh and Liao published in 2016 reported that anti-VZV serum IgM antibody has lower sensitivity but if present is highly suggestive of acute infection [20]; however, in our patient VZV polymerase chain reaction in the cerebrospinal fluid was negative, ruling out a VZV infection. The VZV PCR is highly sensitive and specific [2,3,20].

The management of multiple cranial neuropathy palsies relies on accurate diagnosis with specific therapy aimed at the underlying cause [2]. A lack of clear etiology represents a management challenge and specific recommendations cannot be made. Empiric therapy with corticosteroids seems a logical management option [2,15]. The etiology cause of our patient was then finally categorized as idiopathic.

## Conclusions

This is the youngest patient ever reported with recurrent polyneuritis cranialis of idiopathic etiology with a very odd sequence of cranial nerve palsy affecting the right VI, left V, left VII and left IV cranial nerves without prominent associated symptoms. GBS, sarcoidosis, Lyme disease, VZV, myasthenia gravis, ophthalmoplegic migraine, masses' infiltrative processes should remain in the differential. The outcome was excellent despite the lack of specific therapies suggesting a benign course. Clinicians must be aware of this rare entity to avoid unnecessary testing and to provide a more effective diagnosis and counseling to patients. As is usual in case reports, more cases are needed to characterize this unique clinical diagnosis.
